# Supplementary material for: An approachable, flexible and practical machine learning workshop for biologists
Source: Bioinformatics. 2022 Jun 27;38(Suppl 1):i10–8. doi: 10.1093/bioinformatics/btac233 (PMC9236579; doi:10.1093/bioinformatics/btac233)
Supplement: btac233_Supplementary_Data [file btac233_supplementary_data.pdf]

Supplementary material for: An approachable, flexible, and practical machine learning workshop for biologists

Chris S Magnano, Fangzhou Mu, Rosemary S Russ, Milica Cvetkovic, Debora Treu, Anthony Gitter

Supplementary Tables

Table S1. Participants of the three workshops. The single "Other" response was noted as "Research Specialist/Technician". The table includes participants who completed the pre-survey but did not complete the assessment or post-survey.

Workshop date	Undergraduate	Graduate	Post-doctoral	Staff Scientist	Principal Investigator	Other	Total
May	0	2	5	2	0	1	10
August	2	10	5	0	1	0	18
September	0	9	6	4	0	0	19
Total	2	21	16	6	1	1	47

Table S2. In-workshop assessment results. The numbers presented in this table represent the number of open-ended responses to assessment questions that were coded along each of the dimensions listed. Total number of responses per concept differ because not all respondents answered all of the questions.

Target ML concept represented in different assessment questions	Coded response types	Responses
Experimental design	Correctly identified class label	31
	Incorrectly identified class label	1
	Correctly identified number of instances	28
	Incorrectly identified number of instances	4
	Correctly identified model	31
	Incorrectly identified model	1
	Correctly identified evaluation metrics	30
	Incorrectly identified evaluation metrics	2
	Correctly identified data split	31
	Incorrectly identified data split	1
Data leakage	Correct identification	14
	Incorrect identification	11
	Unsure	4
Overfitting	Provided justification by noticing gap in training and test sets	10
	Other correct justification	5
	Incorrect justification	2
	Provided no justification	11
	Unsure	3
Performance metrics	Correctly identified metrics as appropriate (no justification)	24
	Correctly identified metrics as appropriate and mentioned additional appropriate metric	3
	Correctly identified metrics as appropriate and mentioned most important confusion matrix quadrants	2
Trust	Results are valid	14
	Results are invalid (no explanation)	7
	Results are invalid because of data splitting	5
	Results are invalid because of performance gap in training and test sets	2
	Unsure	1
